# Machine learning based approach to intrusion detection in internet of things environments

**DOI:** 10.3389/frai.2026.1760137

**Published:** 2026-04-17

**Authors:** Oluwatoyin Esther Akinbowale, Adebola Tajudeen Adesina, Mulatu Fekadu Zerihun, Polly Mashigo

**Affiliations:** 1 Faculty of Economics and Finance, Tshwane University of Technology, Pretoria, South Africa; 2 Department of Mechatronics Engineering, Bells University of Technology, Ota, Nigeria

**Keywords:** cybersecurity, intrusion detection, IoT, machine learning, networks

## Abstract

The growing security requirements of Internet of Things (IoT) networks where heterogeneous networks and resource-constrained devices offer exponentially increased attack surface, was addressed using machine learning based intrusion detection system. Open source secondary quantitative IoT intrusion traffic data was obtained and trained using machine learning models. The dataset comprises of over one million labeled flow records consisting of 34 kinds of attacks and benign traffic. First, extensive preprocessing including managing of missing values, encoding features, scaling, and removal of redundancy was carried out followed by the training of three supervised machine learning (ML) classifiers namely Decision Tree (DT), Random Forest (RF), and Support Vector Machine (SVM) for the differentiation of the different types of intrusions. The performance evaluation of the ML models was conducted by evaluating the accuracy, precision, and recall, and F1-score. It was observed that Decision Tree model was the most outstanding in terms of overall accuracy (99.36%) and respectable performance in prevalent attack classes, and was closely followed ccy Random Forest (99.27%) while SVM lagged behind with an accuracy of 80.08% due to computational constraints in handling massive amounts of big data. Inter-arrival time and total packet size were identified as the significant discriminators in malicious behavior through feature-importance analysis. Conclusively, the tree-based models, and specifically Decision Trees, offer extremely effective and interpretable solution for real-time IoT intrusion detection, and provide future avenues in handling class imbalance and examining lightweight, ensemble, and deep-learning approaches for robust detection of rare and unknown threats. This study contributes to cybersecurity via the identification and classification of intrusions in IoT devices for proper mitigation.

## 1 Introduction

In the current era of technology, Internet of Things (IoT) continues to gain traction, due to increasing use being an integral part of everyday life, IoT was initially named in 1999 by British technologist Kevin Ashton. IoT refers to an array of devices that have access to the internet and even can “speak” with each other. Due to this IoT envisioned a world where there was an enormous universe of devices communicating with one another and gathering and processing data such that they could perform tasks independently like Pushing beyond current uses just like process automation, home appliance automation, intelligent cars, smart grids, health care system, decision analytics, educational advancement, industrial development. IoT consists of many elements, such as sensors, some actuators, different software, processors, and other types of technologies employed to link and facilitate devices and systems to exchange data over the Internet. Who would have ever imagined a notion that seemed to be science fiction today is an imminent reality in a rapid phase (Ashton, 2009). According to the Statista report, there is a projection that the number of IoT based devices in the world would increase by 50% from 15.9 billion in 2023 to over 32.1 billion in 2030 (Statista Report, 2024). With all the benefits of IoT, cybercriminals have a wider attack surface because of the vast number of devices that are interconnected, most of which lack strong security controls and lack much processing power (Symantec, 2023). As certain recent studies have shown, more than 60% of IoT attacks target weaknesses like old firmware, weak authentication mechanisms and unsafe network protocols (Al-Hadhrami and Hussain, 2020). Though IoT networks continue to be vulnerable to ransomware, botnets, Distributed Denial of Service (DDoS) attacks, man-in-the-middle (MitM) attacks, and many more types of cyberattacks. The heterogeneity resulted due to merging various nodes in IoT is vulnerable to a serious security breach. Making the resource-constrained IoT nodes strongly secure is the greatest challenge since such nodes are most vulnerable to cyberattacks as they have constrained resources. Such nodes’ vulnerable by nature is easily accessed by malwares that compromise the integrity of the whole network. Intrusion detection systems are primitive but potent security devices for making IoT-embedded environments secure (Islam et al., 2021). Conventional security appliances like firewalls, access control, and encryption are insufficient to protect them. They do not work and are inefficient on large networks having numerous devices, with each device being a vulnerability point. The IoT network can be better secured by the inclusion of the intrusion IDS, the second defense against security attacks (Adewole and Torra, 2023). The distinctiveness of intrusion detection systems is that it is capable of determining whether the source of this data packet is a legitimate user or an attacker. They are excellent at passively sniffing traffic and listening, looking, and sensing live data packets. In most cases, intrusion detection systems are network-based or host-based depending on the physical point of deployment within the system. Early detection of malicious activity via monitoring of network traffic is crucial to, intrusion detection technologies must be enhanced and developed in a hurry owing to the increase of advanced cyber anomalies. Most notable intrusion detection challenge is detecting advanced cyberattacks amidst typical network traffic.

Thus, development and massive market for IoT technology has been created that generated its economic value, and drew many people - from scientists to inventors, entrepreneurs to businesspeople, and even hackers. IoT even spawned other schemes of investment on technological innovation on businesses. Innovators and inventors keep evolving and selling fresh IoT applications that further increase convenience and efficiency for a vast majority of activities. The widespread use, however, has also invited vulnerabilities in IoT networks on several occasions in security breaches, which are also employed by hackers in finding IoT device vulnerability weaknesses. IoT platform has proven to be a lucrative cyberattack target due to businesses emerging at lightning speed through IoT as well as financial incentives.

It has also led to the serious escalation of security threats caused by IoT because of rising numbers of connected devices and their integration into critical infrastructures. Open-ended vulnerabilities have been exploited by attackers when crafting complex cyberattacks, poor encryption, and poor authentication as they sought to execute successful attacks. This ever-growing demand calls for strong and improved security controls, real-time threat detection, and continuous innovation in intrusion detection technologies to defend IoT ecosystems. Machine learning (ML) has also emerged as a viable solution to address the issue of intrusion detection in IoT networks because it can identify deviations and anomalous patterns in the network traffic (Telnyx, 2024).

ML-based intrusion detection system in IoT devices have been revised in prominent literature. Ensemble-based techniques, in this context, have become very popular since they can advance the execution and reliability of IDS. Abbas et al. (2022), for instance, recommended an ensemble IDS model that incorporates a lot of ML techniques such as Naive Bayes (NB), DT, and logistic regression (LR). The execution of the model in detecting malicious network traffic and it was established when it was tested against the CICIDS2017 intrusion detection dataset and achieved 88.96% accuracy for multi-class sorting and 88.92% accuracy for binary classification. Likewise, Awotunde et al. (2023) suggested ensemble approaches using modern state-of-the-art machine learning algorithms like AdaBoost, Random Forest, Bagging, XGBoost, and Extra Trees. Their method utilized the ToN-IoT dataset, which contains telemetry data of seven IoT devices: refrigerator, thermostat, modbus, motion light, GPS tracker garage door, and weather sensors. Their ensemble models utilized heterogeneous sources of data to improve detection accuracy and generalizability across a vast range of IoT environments. Due to the intrusion detection issue in IoT networks, ML has also been proposed as a useful solution because it can detect anomalies and unusual network traffic behavior. Some of the relevant research works in the literature have considered ML-based intrusion detection in IoT networks. Ensemble-based techniques are particularly of interest in this regard because they can assist in making IDS more effective and efficient.

Subsequently, a superior prediction-performing AdaBoost ensemble algorithm was then trained on feature-cleaned features. Two of the largest IDS datasets, NSL-KDD and BoT-IoT, have been utilized by their paper in an attempt to legitimize the model as well as show its success at maximizing IDS efficiency. They report an increase in applications of ML-empowered ensemble methods for IoT network hardening that offers adaptive and scalable defensive measures against evasive cyber-attacks.

The Internet of Things is expanding at an astounding rate, with an estimated 40 billion connected devices by 2030 (Awotunde et al., 2023). Due to their intrinsic security flaws, IoT devices are extremely susceptible to attack, and their proliferation is creating difficult security problems. The most notable are: repeated attacks according to NETGEAR (2024), home network devices subject to roughly ten typical cyberattacks every day, sector weaknesses more than 0.7 times as many industrial firms experienced cyberattacks on IoT devices (Kaspersky, 2023). In terms of data breaches in 2023, healthcare analytics breaches primarily caused by compromised IoT devices affected 25% of Americans (Asimily, 2024). Unencrypted Data Asimily (2023) reports that 98% of Internet of Things traffic is unencrypted, making it possible to target data.

Conventional intrusion detection systems (IDS) are not appropriate for secure IoT environments. Because it is rule-based, it produces a high false positive rate and is ineffective against novel attacks, particularly in dynamic and resource-constrained environments. Therefore, an IDS that is machine learning based and adaptive is required in order to recognize and react to emerging threats in IoT networks.

With the exponential growth of IoTs to an estimated 40 billion devices by 2030, convenience came at a cost in the form of increased security vulnerabilities (Awotunde et al., 2023). Standard attacks like DDoS and data breaches take advantage of exposure because 98% of IoT traffic is not encrypted (Hazman et al., 2024) and the majority of devices run outdated software (Al-Hadhrami and Hussain, 2020). Due to resource and scalability limitations, the Internet of Things poses a threat to traditional intrusion detection systems (IDS) (Al-Hadhrami and Hussain, 2020). Rather, through dynamic anomaly detection, machine learning offers a practical solution (Hazman et al., 2024).

The aim of this study is to apply ML based approach to security system in IoT devices.

Select and implement three existing machine learning model - DT, SVM and RF for intrusion detection on the IoT Intrusion Detection Dataset from Kaggle (IoT Intrusion Detection).

- Evaluate the accuracy of the recall, precision, and F1-score for each model.
- Compare each outcomes and recommend the top-performing model for IoT-based intrusion detection systems.

With more and more IoT devices coming into consumer as well as industrial markets, this study is significant in the manner in which it may bring security into these categories of devices. It has been estimated that there will be approximately 40 billion IoT devices by the year 2030 (Al-Hadhrami and Hussain, 2020), to which there poses a vital risk in the form of cyberattacks. According to recent statistics, the home network devices experience 10 cyber-attacks per day (NETGEAR, 2024) and must be protected well. The traditional rule-based IDS is not suitable for the dynamic and resource-scoped IoT scenario (Awotunde et al., 2023). They are also not able to learn new attacks and it is computationally expensive. Machine learning offers an economical and dynamic method for the detection of intrusion and anomalies (Al-Hadhrami and Hussain, 2020). DT, SVM and RF, current ML algorithms, are compared in this research work to detect IoT network intrusion based on Kaggle’s IoT Intrusion Detection Dataset (IoT Intrusion Detection). Comparative studies will assist scholars and professionals make extra educated decisions approximately what model is best for IoT security. Intrusion detection is the basis for preventing data protection and physical harm, such as unauthorized physical entry into buildings through hijacked smart locks or endangering patients’ lives through hijacked medical equipment. The studies can drive product development on the vendors’ end and guide policymakers. Overall, the study is filling an extremely vital void, opening up safe havens for security software and IoT.

This study concentrates on implementing and evaluating existing ML models for detecting network-level intrusions in IoT systems using the IoT network intrusion detection dataset. It involves selecting three supervised learning models DT, SVM and RF implemented with Python’s scikit-learn library.

## 2 Literature review

### 2.1 Theories related to intrusions, cyberattacks, and cybersecurity

Theoretical models play a crucial role in cybersecurity as they describe the why, how, and impact of cyber threats. Below are five of the most relevant theories related to intrusions, cyberattacks, and cybersecurity that relates to his study.

#### 2.1.1 Deterrence theory

Deterrence theory, rooted in criminology, dictates that punishment will discourage undesirable behavior (Gibbs, 1975). In cyber space, it would imply deterring attackers through legal, economic, or reputational punishment. The theory is used in policy-making like the Budapest Convention on Cybercrime, which dictates punishment for cybercrime (Council of Europe, 2001). However, its use is still limited because of attribution and anonymity problems in cyberspace.

#### 2.1.2 Routine activity theory

Routine Activity Theory (RAT) requires that crime occurs when an offender, a suitable target, and the absence of a capable guardian converge (Cohen and Felson, 1979). In terms of cyberattack, this means that cybercriminals attack vulnerable systems with weak defenses. RAT asks why particular systems are attacked (whose vulnerabilities are revealed) and calls for protection, i.e., intrusion detection systems, to prevent attacks.

#### 2.1.3 Game theory

Game Theory is the analysis of strategic interaction among rational players (von Neumann and Morgenstern, 1944). In cyber security, it is used to model defender and attacker behavior as a strategic game. It predicts attack patterns and optimizes the utilization of defense resources, in particular against advanced persistent threats (APTs). Game-theoretic models are being widely used in the design of resilient security architectures.

#### 2.1.4 Social engineering theory

Social Engineering Theory analyzes how psychological manipulation takes advantage of human vulnerability in order to obtain unauthorized access (Mitnick and Simon, 2002). Phishing and pretexting are common practices. The theory identifies the human element as a weakness in cybersecurity that necessitates user education and awareness programs to avert attacks that bypass technical security.

#### 2.1.5 Risk management theory

Risk Management Theory addresses the identification, analysis, and reduction of risk (Stoneburner et al., 2002). In cybersecurity, this is addressed through threat analysis and the implementation of controls like encryption and access controls. This theory forms the foundation of frameworks like the NIST Cybersecurity Framework that enables organizations to prioritize resources accordingly and protect assets. The practical application of each of these theories is as follows: The Deterrence Theory guides legal frameworks to combat cybercrime while the RAT guides reduction of vulnerability and enhancement of security. The Game Theory guides strategic defense policy while the Social Engineering Theory supports user training programs. Finally, the Risk Management Theory guides overall cybersecurity strategies.

With these theories in place, an interdiscipline model can be created to address the dynamism of cyber threats. These theories are a solid basis for addressing and understanding intrusions, cyberattacks, and cybersecurity concerns. United in knowledge, researchers and practitioners can conceptualize better ways of strengthening security in a growing digitalised domain.

### 2.2 Machine learning implementations in IoT IDS

This section discusses some of the ML models employed for intrusion detection in IDS. Existing authors such as Kim et al. (2018), Hasan et al. (2021), Khan et al. (2021), and Saranya et al. (2020) reported on the potentials of machine and deep learning for intrusion detection in IDS. Specifically Khan et al. (2021) employed the Recurrent Neural Network (RNN) deep learning-based technique for intrusion detection in Message Queuing Teletry Transport (MQTT) Enabled IoT. The result obtained indicated that the DNN achieved accuracies of 0.9992, 0.9975, and 0.9494 for accuracies for uni-flow, Bi-flow, and Packet-flow, respectively for binary classification. Nevertheless, there was a reduction in the accuracies to 0.9708, 0.9812, and 0.9079, respectively. On the overall the DNN achieved an accuracy of 97.13%, thereby outperforming other models such as the Long Short Term Memory (LSTM) and Gated Recurrent Unit (GRU).

Akinbowale et al. (2025) employed deep learning models specifically the Convolutional Neural Network (CNN) and LSTM for cyberfraud incidence classification and time series prediction using secondary dataset obtained from the reports of the South African Banking Risk Information Centre (SABRIC). The results indicated that both models excelled in the classification assignment, however, the LSTM model with an accuracy of 96.80% outperformed the CNN which has an accuracy of 96.17%.

Despite the robust performance of machine and deep learning models in intrusion detection, Ieracitano et al. (2020) reported that a novel statistical analysis and autoencoder developed for intrusion detection outperformed the conventional machine and deep learning models in classifying intrusions.

Sharafaldin et al. (2018) indicated that the robustness of intrusion detection system is a function of the data used for training the model. The study contributed a benchmark, modern and practical dataset for intrusion detection and machine learning studies.

#### 2.2.1 Decision trees

Decision Trees (DTs) are widely used in IoT traffic attack pattern detection since they are very interpretable. Their tree structure and simple decision rules enable cybersecurity experts to view exactly what features trigger an alarm, making it extremely easy to understand the model’s reasoning. Such transparency is essential in identifying subtle attack patterns in advanced IoT settings since it enables effective debugging, model calibration, and planning. DTs also enable high-speed processing of heterogeneous sensor data and are therefore particularly suitable for real-time threat detection within resource-constrained IoT systems. Explainability not only enables trust in the auto-detection mechanism but also enables quick response to attacks and continued system refinement.

Aminanto and Kim (2018) defended against Wi-Fi impersonation attacks through DT in IoT networks with 89% of F1-score but were limited for handling high-dimensional data.

Al-Fawa’reh et al. (2023) employed an accuracy decision tree of 87% to BoT-IoT and accounted for class imbalance as the major defect, especially in the case of very rare types of attacks like data theft.

#### 2.2.2 Random Forest

Random Forest is a technique with numerous decision trees being employed to enhance overall accuracy and avoid overfitting by leveraging combined predictions from numerous models. The technique enhances precision and performance through processes such as bootstrap aggregation and random feature selection, eliminating noise and variability of information. Where one has to work with massive amounts of IoT data—high-dimensional and streaming—Random Forests excel by distributing the computational workload across many trees and parallel processing capabilities. Through rapid and balanced aggregation of decision outputs, they are best positioned for concurrent monitoring, outlier detection, and predictive maintenance applications in IoT environments, where diversity and volume of data require robust and scalable solutions. This combination of precision, speed, and adaptability has made Random Forest popular with research and business circles alike as a first choice for processing and analyzing gigantic-scale IoT data.

Awotunde et al. (2023) employed RF to ToN-IoT with a correctness of 94% via feature selection to decrease computation overhead. They reported the ability of RF in the handling of heterogeneous IoT data but demonstrated trade-offs in model interpretability.

Diro and Chilamkurti (2018) applied a distributed RF model to edge devices for IoT and resulted in 91% detection rates for DDoS attacks. They cautioned against unnecessary memory usage on low-end devices.

#### 2.2.3 Support vector machines (SVM)

Support Vector Machines (SVMs) are capable of dealing with high-dimensional problems because they are able to find best-separated hyperplanes and are also able to use the kernel trick while mapping the data onto higher feature spaces. SVMs are actually great when performing classifications when there are a large number of features relative to samples. However, the computational cost of SVMs grows exponentially with the dataset size, which in real-time IoT applications constantly generating large volumes of data is a problem. In such situations, the training and inference steps become the bottlenecks, and they determine the scalability and responsiveness of SVMs.

Abiramasundari and Ramaswamy (2025) tested SVMs on CICIDS2017 and attained 88% accuracy for DDoS attack detection. They used kernel tuning to attain precision and computational overhead.

#### 2.2.4 Ensemble methods

Hybrid approaches are increasingly gaining attention in order to counter challenges of IoT data like data skewness. For instance, Abbas et al. (2022) combined RF and DTs over CICIDS2017 with 89% multi-class accuracy. They found ensemble methods to be resistant to overfitting but warned against increased training time, Koroniotis et al. (2019) used gradient boosting on BoT-IoT and attained a detection accuracy level of 93%. Hybrid models need to be utilized, as per them, to address the “cold start” problem of IoT when there is no history for the newly added devices.

Abbas et al. (2022) also reported on the development of a novel Ensemble-based technique for intrusion detection system in IoT. The outcome of the study indicated significant improvement with respect to binary and multi-class type classification compared to existing model.

Table 1 presents an overview of selected empirical researches and their key findings.

**Table 1:** Overview of selected empirical researches and their key findings.

Author(s)	Dataset model performance	Key findings
Aminanto and Kim (2018)	Wi-Fi Decision Tree	Achieved 89% F1-score. Effective against impersonation but limited by dimensions.
Al-Fawa’reh et al. (2023)	BoT-IoT	Decision Tree achieved 87% accuracy. It addressed class imbalance but struggled with rare attacks.
Awotunde et al. (2023)	ToN-IoT	Random Forest achieved 94% accuracy. Feature selection reduced overhead but less interpretable
Diro and Chilamkurti (2018)	Edge distributed RF	Achieved 91% detection rate.
Abiramasundari and Ramaswamy (2025)	CICIDS2017	SVM achieved 98.7% accuracy. Has Radial Basis Function for effective kernel tuning, but computationally intensive.
Abbas et al. (2022)	CICIDS2017	Ensemble (RF + DT) achieved 89% accuracy. It is robust but slow training.
Koroniotis et al. (2019)	BoT-IoT Gradient	Boosting gradient achieved 93% accuracy. It is addressed cold start, but hybrid models needed.
Zhang et al. (2025)	ToN-IoT	The proposed method “two-stage feature selection and Bayesian optimization” achieved an accuracy of 97.22%.

Most of the literature (e.g., Aminanto and Kim, 2018; Abiramasundari and Ramaswamy, 2025) use non-IoT datasets like CICIDS2017 or NSL-KDD that do not represent IoT-specific attacks like firmware exploits or sensor spoofing. Furthermore, comparative research is scarce in which DT, RF, and SVM are benchmarked on a single IoT dataset comprehensively. For example, Awotunde et al. (2023) evaluated RF on ToN-IoT only, not against DT or SVM. In addition, real-world problems such as class imbalance (Al-Fawa’reh et al., 2023), latency of real-time detection (Diro and Chilamkurti, 2018), and computational complexity (Al-Hadhrami and Hussain, 2020) are still pending.

By comparing the performance of Decision Tree (DT), Random Forest (RF), and Support Vector Machine (SVM) on the Kaggle IoT dataset for intrusion detection systems, this study identifies the most feasible algorithms for use in IoT intrusion detection.

## 3 Methodology

### 3.1 Research design

This study employs secondary quantitative dataset specifically the intrusion detection dataset on Kaggle (2023), with network traffic data from seven IoT devices, labeled as “normal” and “attack.”

**Figure 1:**
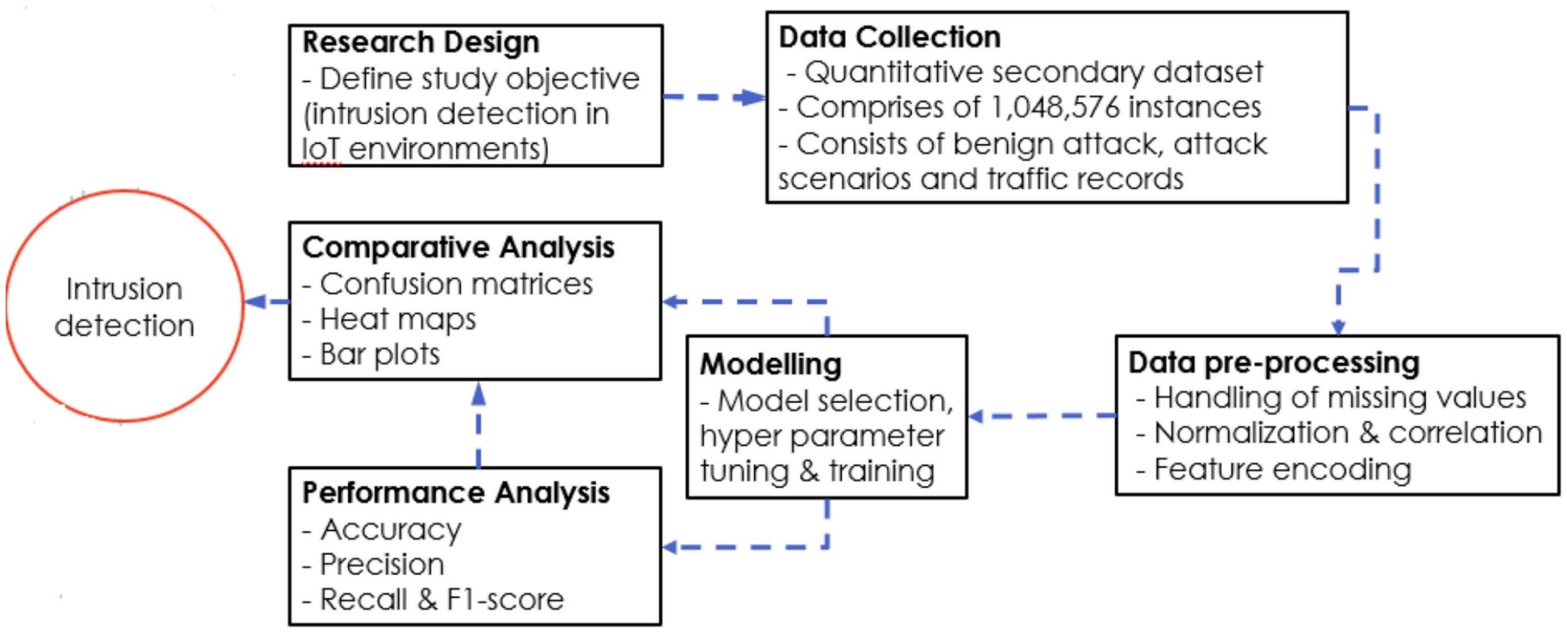
Research design.

The details of the research design is shown in Figure 1.

### 3.2 Data set and modelling

The dataset, consisting of 1,048,576 instances, includes benign traffic as well as a number of attack scenarios, and community traffic records. It includes a diffusion of features such as flow-based metrics (e.g., flow duration, header length), protocol signs (e.g., HTTP, TCP, UDP), flag-based features (e.g., SYN, ACK flags), and statistical measures (e.g., mean, standard deviation). The dataset labels categorize traffic into various attack types (e.g., DDoS, DoS, Mirai botnet attacks) and benign traffic, simulating realistic IoT network scenarios suitable for developing and assessing intrusion detection systems. The IoT intrusion detection dataset is well-suited for demonstrating intrusion detection because of its complete illustration of IoT network traffic, including diverse attack types and benign activities. Its size and feature variety enable effective model training and testing, although preprocessing is necessary to handle missing values and feature correlations, ensuring robust model performance. Furthermore, the dataset provides a good testing ground for model testing. For handling class imbalance within intrusion detection datasets, SMOTE (Synthetic Minority Over-sampling Technique) was applied to the training data after the train (80%)–test (20%) split excluding the test set to avoid data compromise during model’s evaluation.

Moreover, feature scaling was utilized to ensure all features have balanced influence in the model’s learning process so that the performance and generalizability of the models improve. The ML models specifically Random Forest Decision Trees, Support Vector Machine (supervised learning) were implemented in the Python environment. Some of the tools and technologies are embedded in the Python, scikit-learn library for execution and evaluation (Pedregosa et al., 2011). Assessment metrics employed consist of accuracy, precision, recall, F1-score and some of the procedural steps undertaken include statistics preprocessing, version training, performance evaluation, comparison. Some of the excluded activities include developing new algorithms, hardware security, real-world deployment, real-time detection. Using the IoT intrusion detection dataset on Kaggle (IoT intrusion detection), this study utilises existing ML models for intrusion detection and compares them in a quantitative studies design. The design is composed of several phases: First, the data is loaded and assessed for structure and features. Data preprocessing includes missing value management, scaling of features, and encoding categorical features using Python library scikit-learn. The data is split into 0.800 training data and 0.200 test data. The training data is trained with three ML models DT, SVM and RF with hyperparameter tuning done using GridSearchCV. The test data performance is verified based on performance measurements like precision, recall, F1-score and accuracy. The models are evaluated to determine the model with the best performance, and visualizations such as confusion matrices are utilized to make sense of performance. All process steps are recorded, giving an accurate description of the research process.

The IoT network intrusion detection dataset, downloaded from Kaggle, provides a robust foundation for intrusion detection system analysis. It comprises 46,686,579 network-traffic records—1,048,576 of which were sampled for detailed study each described by 46 flow-based, protocol, flag, and statistical features. These records fall into 34 labeled categories: one benign (normal) class and 33 distinct attack classes. Their frequencies range from large-scale volumetric attacks, such as DDoS–ICMP Flood (161,281 to several million instances), to highly infrequent application-level exploits like Uploading_Attack (just 23 samples). Table 2 illustrates all attack types with the number of occurrences. Such high class imbalance in which common attacks vastly outnumber minority classes reflects real-world IoT traffic and denotes the necessity of careful management and evaluation methods to ensure minority class detection performance.

**Table 2:** Attack types and the number of occurrences.

Attack Type	Description	Instance Count
DDoS-ICMP_Flood	Internet control message	30,662
DDoS-UDP_Flood	User datagram protocol flood	23,085
DDoS-TCP_Flood	Distributed denial of service - transmission control protocol	19,478
DDoS-SYN_Flood	Synchronize flood	17,688
DDoS-PSHACK_Flood	Push acknowledge flood	17,569
DDoS-RSTFINFlood	Reset finish flood DDoS	17,088
DDoS-SynonymousIP_Flood	Synonymous internet protocol DDoS	15,432
DoS-UDP_Flood	User datagram protocol flood DoS	14,390
DoS-TCP_Flood	Transmission control protocol flood DoS	11,310
BenignTraffic	Normal traffic	4,571
Mirai-greeth_flood	Generic routing encapsulation Ethernet flood	4,251
Mirai-udpplain	Mirai user datagram protocol plain	3,792
Mirai-greip_flood	Mirai generic routing encapsulation internet protocol flood	3,266
DDoS-ICMP_Fragmentation	Internet control message DDoS	1954
MITM-ArpSpoofing	Man-in-the-middle - address resolution protocol spoofing	1,372
DDoS-UDP_Fragmentation	Distributed denial of service - user datagram protocol	1,218
DoS-SYN_Flood	Synchronize flood DoS	8,645
DNS_Spoofing	DNS spoof	743
Recon-HostDiscovery	Host discovery	579
Recon-OSScan	OS scan	413
Recon-PortScan	Port scan	373
DoS-HTTP_Flood	Hypertext transfer protocol flood DoS	333
VulnerabilityScan	Vulnerability scan	148
DDoS-ACK_Fragmentation	Acknowledge fragmentation DDoS	1,174
DDoS-HTTP_Flood	Hypertext transfer protocol flood | Flood DDoS	109
DDoS-SlowLoris	Slow low-rate internet attack DDoS	94
DictionaryBruteForce	Dictionary Brute force	57
SqlInjection	Structured query language injection	32
BrowserHijacking	Browser hijack	23
CommandInjection	Command injection	23
XSS	Cross-site scripting	15
Backdoor_Malware	Backdoor malware	10
Recon-PingSweep	Ping sweep	10
Uploading_Attack	Upload attack	3

**Figure 2:**
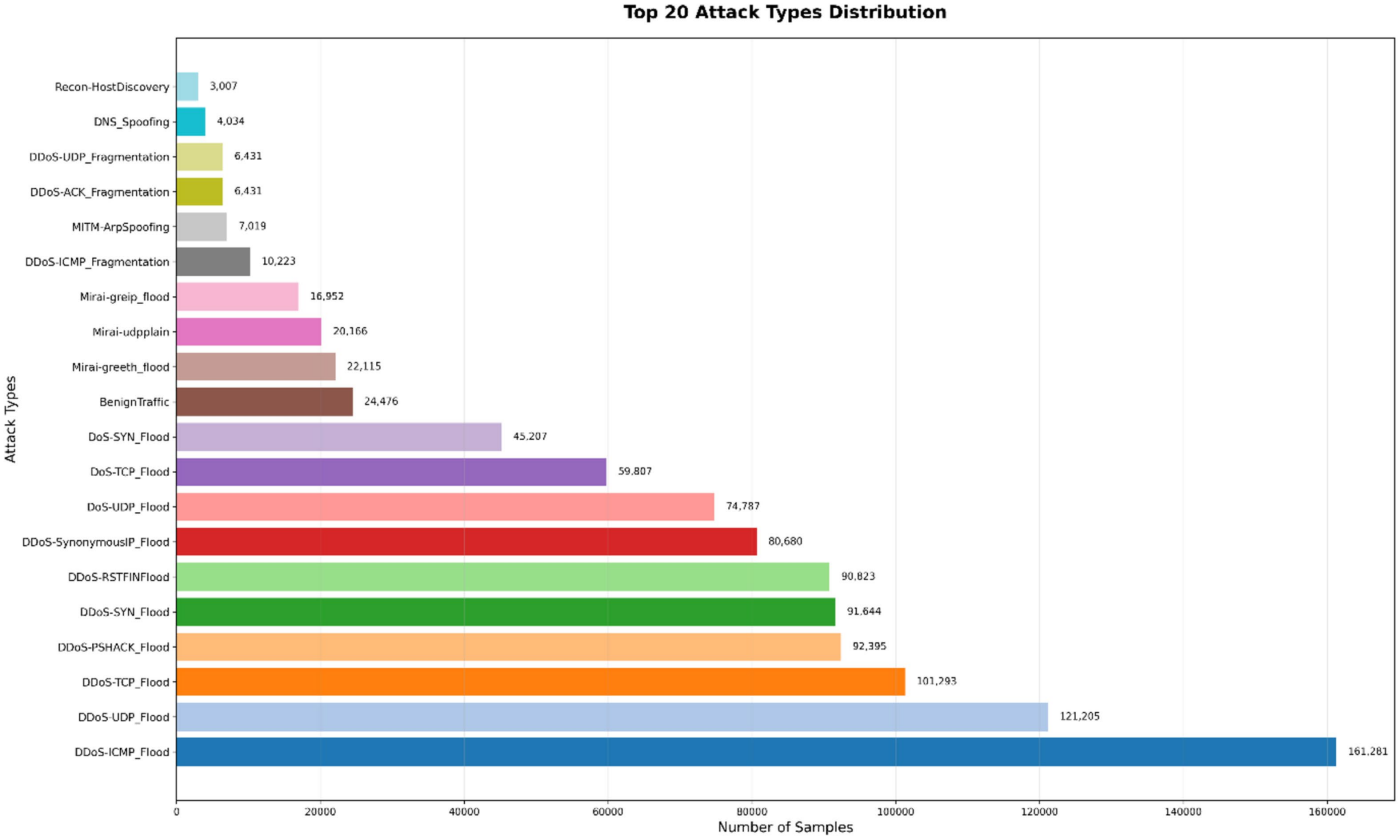
The detailed distribution of attack types.

Prior to training, raw data was subjected to rigorous cleaning and conversion. The primary preprocessing operations included data cleaning, feature encoding, scaling, and feature reduction. For example, infinite and missing values were identified and handled (by deletion or imputation of a value) and redundant records removed. Categorical labels (attack type names) were converted into numeric codes to make them ML algorithm compatible. Any feature with a predominant proportion of missing values was deleted to reduce the dataset to its bare essentials. The feature scaling was thereafter done: numerical attributes were normalized (e.g., by standardisation or min–max scaling) in an effort to avoid one feature dominating others due to scale. Features of zero variance were eliminated finally to focus on descriptive attributes. The following were undertaken as part of the pre-processing activities:

- Removal of missing values in the dataset features during Missing Value Handling.
- Dropping of constant or nearly constant features that provide no informational value during Constant/Redundant Feature Removal.
- In feature scaling (Normalization), scaling methods such as min max normalization or z score standardization was used to ensure numerical features contribute equally.
- Correlation analysis was achieved by computing the feature correlation matrix and, in case features are highly correlated, applying dimensionality reduction (e.g., Principal Component Analysis) to address redundancy.
- Records dividing the preprocessed dataset changed into a training subset and test checking out subset with a typical 0.800 (training dataset) to 0.200 (testing dataset) split.
- Categorical Encoding Categorical features were encoded using LabelEncoder, assigning a unique integer to each category to enable numerical processing.
- Constant Feature Removal Features with zero variance (constant values) were removed, as they do not contribute to model learning.
- Highly Correlated Feature Removal pairs of features with a correlation coefficient greater than Pearson correlation coefficient of 0.95 were eliminated to reduce redundancy, multicollinearity and to enhance model efficiency. In the process a total of 14 features were removed due to their lower variance contributions while others (20 features) with domain relevance having attributes with higher relevance to network intrusion detection were retained. The retained features (displayed in Figure 2) are categorized into four groups, reflecting different aspects of network traffic (a) Flow-based features: This metrics describe the characteristics of network flows, capturing temporal and size-related properties (b) protocol features: Indicators for identify the communication protocols being used (c) flag features: Counts and indicators for Transmission Control Protocol (TCP) flags, highlight control signals in TCP connections.

### 3.3 Machine learning model development

#### 3.3.1 Model selection

The following supervised models are selected because they are used as per the IoT Network Intrusion Detection Dataset dataset:

- Decision Trees: For its sake being easy to understand and explain to others, even if it was used only as a reference model (Khan et al., 2021).
- Support Vector Machine (SVM): High-dimensional and non-linear features are generally difficult to memorize, but the kernel functions are used for this purpose. It is the main reason why support vector machines were implemented in the first place. Among which were the highlights of the latest research studies that have been brought up in this cybersecurity domain such as IoT network intrusion detection.

#### 3.3.2 Model training and evaluation

The models were developed on the preprocessed training data, with hyperparameter tuning achieved the usage of grid search with three-fold cross-validation to perceive ultimate parameters. The parameters employed for the model’s training is presented in Table 3.

**Table 3:** Model hyperparameters.

Model	Key parameters
Random Forest	n_estimators = 100, max_depth = 20, min_samples_split = 5, min_samples_leaf = 2, n_jobs = −1
Decision Tree	max_depth = 25, min_samples_split = 10, min_samples_leaf = 4
Support Vector Machines (SVM)	kernel = ‘rbf’, C = 1.0, gamma = ‘scale’, probability = True

The Random Forest model has 100 trees to enhance accuracy, and reduce computation speed. It has a maximum depth of 20 to limits the tree complexity to avoid overfitting and minimum samples split of 5 to limits splits to prevent over-specificity. The second model (Decision Tree) has a maximum depth of 25 to restrict complexity and maintain simplicity, minimum samples split of 10 as well as minimum samples leaf of 4 to specifies the minimum data needed for prediction. The third model, the Support Vector Machine (SVM) has a regularization (C) factor of 1.0 to compromise fit for simplicity and Kernel (gamma) to tune the flexibility to non-linear data.

For SVM, a stratified subset of 50,000 samples was used if the training data exceeded this size to manage computational complexity. The use of the stratified subset for SVM was informed due to the fact that SVM requires a significantly higher computational complexity for training, especially for large-scale datasets. Conversely, decision tree and random forest present lesser computational complexity for large datasets.

To mitigate this challenge, and resulting sampling bias, the stratified sampling was employed to ensure a proportional class distributions in the subset that is reflective of full training dataset used for the decision tree and random forest models.

The 50,000-sample subset obtained using the stratified sampling method for the SVM training was based on class labels. This ensures that the class distribution in the subset is reflective of the class distribution in the full training dataset. Thus, all 34 attack classes were represented in the subset.

Three ML models were trained with optimized hyperparameters using GridSearchCV, as shown in Table 3. The values of the hyperparameter were informed based existing literature on intrusion detection and tree-based ensemble learning such as Lu et al. (2024), Khraisat et al. (2019) and Liu and Lang (2019). Furthermore, the algorithm design guidelines from scikit-learn documentation were also considered coupled with computational feasibility constraints given the dataset size and hardware limitations.

The personal computer (PC) with used for the training has the following specifications:

- Operating System: Windows 11 Pro 64-bit
- Central Processing Unit (CPU): Intel Core i7-1165G7 (4 cores, up to 4.7 GHz)
- Random Access Memory (RAM) of 16 GB DDR4

##### 3.3.3 Performance evaluation of the ML models

The models were evaluated at the test set the use of the following metrics (a) Accuracy The proportion of correct predictions (b) Precision Precision is the percentage of true effective predictions amongst all nice forecasts generated by the model. It indicates how accurate the positive predictions are, showing the share that are truly correct (c) Recall This is the percentage of real positive forecasts amongst all real positive instances. It reflects the model’s potential to detect all applicable effective cases, capturing what number of real positives are diagnosed (d) F1-Score: This is the harmonic imply of precision and recall, providing a balanced measure that accounts for both metrics. It provides a single score useful when both prediction accuracy and detection completeness matter.

Confusion matrices were employed to offer detailed insights into classification performance, including true positives, false positives, true negatives, and false negatives.

The performance evaluation of the developed models was determined evaluation criteria expressed in Equations 1–4.


$$\text{Accuracy}=\frac{\mathrm{TP}+\mathrm{TN}}{\mathrm{TP}+\mathrm{FP}+\mathrm{TN}+\mathrm{FN}}$$ (1)

$$\text{Precision}=\frac{\mathrm{TP}}{\mathrm{TP}+\mathrm{FP}}$$ (2)

$$\text{Recall}=\frac{\mathrm{TP}}{\mathrm{TP}+\mathrm{FN}}$$ (3)

$$F1\;\text{score}=2.\frac{\text{Precison}*\text{Recall}}{\text{Precison}+\text{Recal}}$$ (4)

Where TP represents correctly identified positives, TN denotes correctly identified negatives, FP stands for incorrectly classified positives, and FN refers to incorrectly classified negatives.

### 3.4 Comparative analysis of the performance of the models employed

The three models were evaluated and compared based on the specified metrics. Visualizations were created to facilitate analysis, including (a) Performance comparison plots involving bar plots comparing accuracy, precision, recall, and F1-score across models. (b) Confusion Matrices heatmaps showing the classification performance for each model, highlighting true and false predictions.

The most accurate model was recognized as the top-performing one, with additional insights provided by feature importance analysis for the Random Forest model, if applicable.

The methodology involved a systematic pipeline:

- Data Preprocessing: Handling missing values through median imputation, encoding categorical variables using LabelEncoder, scaling features with StandardScaler, and removing constant and highly correlated features (correlation > 0.95) to reduce dimensionality from 46 to 32 features.
- Data Splitting: It was divided into 0.800 training and 0.200 testing sets, with stratification to preserve the proportion of classes.
- Model Training: Models were trained with hyperparameter tuning using GridSearchCV and 3-fold cross-validation to optimize performance.
- Evaluation: Models were assessed using accuracy, recall, precision and F1-score with additional visualizations like confusion matrices and feature importance plots.

### 3.5 Tools and technologies

Python was applied for processing your report because of the many advantages that are most well-suited for the demands of data analysis and computational science. It is a very expressive programming language with a simple, readable syntax.

## 4 Results and discussion

### 4.1 Statistical analysis

Aggregated metrics such as Total Sum (Tot sum), Minimum (Min), Maximum (Max), Average (AVG), Standard Deviation (Std), Total Size (Tot size), Inter-Arrival Time (IAT), Packet Number (Number), Magnitude, Radius, Covariance, Variance, and Weight were obtained statistically to provide insights into packet sizes and inter-arrival times (Table 4).

**Table 4:** Descriptive analysis of the dataset employed.

Feature	Mean	Standard deviation	Minimum	25%	50%	75%	Maximum
Flow_duration	5.76	296.57	0.0	0.0	0.0	0.104	99435.76
Inter-arrival time (IAT)	83,173,821	17,038,567	0.0	83,071,569	83,124,519	83,343,901	167,639,426
Total size	124.90	242.92	42.0	50.0	54.0	54.06	13,098.0
Protocol type	9.06	8.94	0.0	6.0	6.0	14.26	47.0
Synchronize count (syn_count)	0.33	0.66	0.0	0.0	0.0	0.06	9.69

These categories enable models to capture diverse patterns in network traffic, crucial for distinguishing between benign and malicious activities.

The IAT (Inter-Arrival Time) feature with a mean of approximately 83 million (most likely microseconds) demonstrates packet time variation, which is critical in detecting high-speed DDoS attack. The Tot size feature with a high range illustrates numerous packet sizes, which is useful in identifying anomalous traffic flows.

Figure 2 illustrates the detailed distribution of attack types, highlighting the prevalence of DDoS attacks and the scarcity of minority classes.

### 4.2 Data exploration

**Figure 3:**
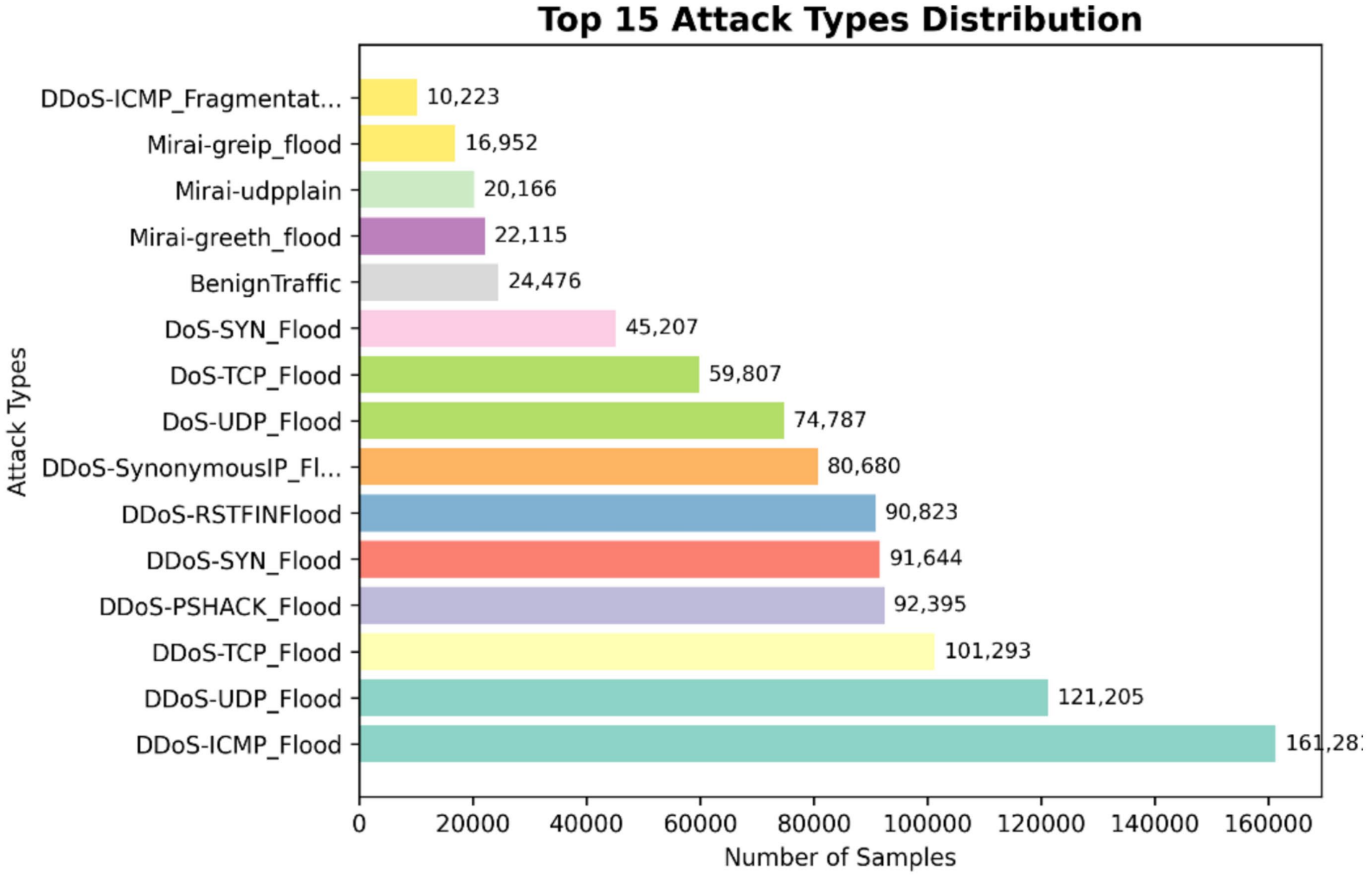
Attack type distribution.

Exploratory data the examination yields insightful data regarding the structure of the data set and informed preprocessing operations. Figure 3 shows a multi-panel plot of salient features of the data. The plot shows the distribution of attack types using a bar plot which validates the frequency of each attack type and confirms class imbalance in the dataset. The provided graph validates the necessity for class imbalance correction to ensure strong and unbiased detection model performance. It shows the distribution of 15 top IoT attacks including the number of samples and frequencies.

**Figure 4:**
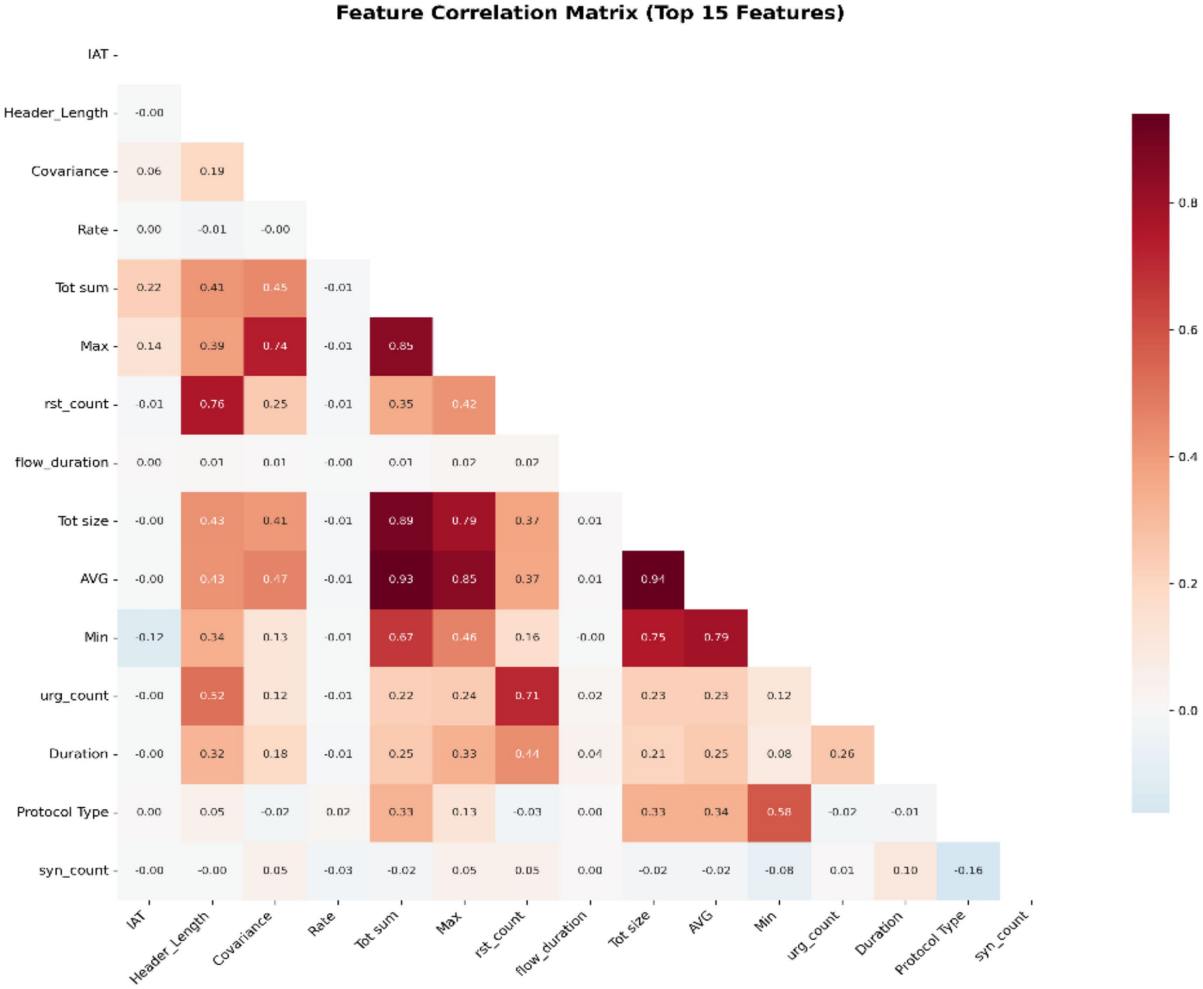
Feature correlation matrix.

A feature correlation matrix, as seen from a heat map in Figure 4, recognises highly correlated feature pairs. Identification and elimination of these redundant features reduce the model’s complexity by removing unnecessary complications and enable simpler interpretation. The area mapped with dark red indicates high positive correlation between the features (from 0.80 to 1), while the areas with light pink indicates low correlation (from 0.00 to 0.20) while the parts with grey colour or near white colour indicates the absence of correlation between the features employed (0.00). The areas mapped with black colour or dark blue colour indicates the presence of negative correlation between the features (below 0.00). The correlation heat map assist in identifying relevant features necessary for intrusion detection and the redundant features. It supports features extraction through reduction in dimensionality.

**Figure 5:**
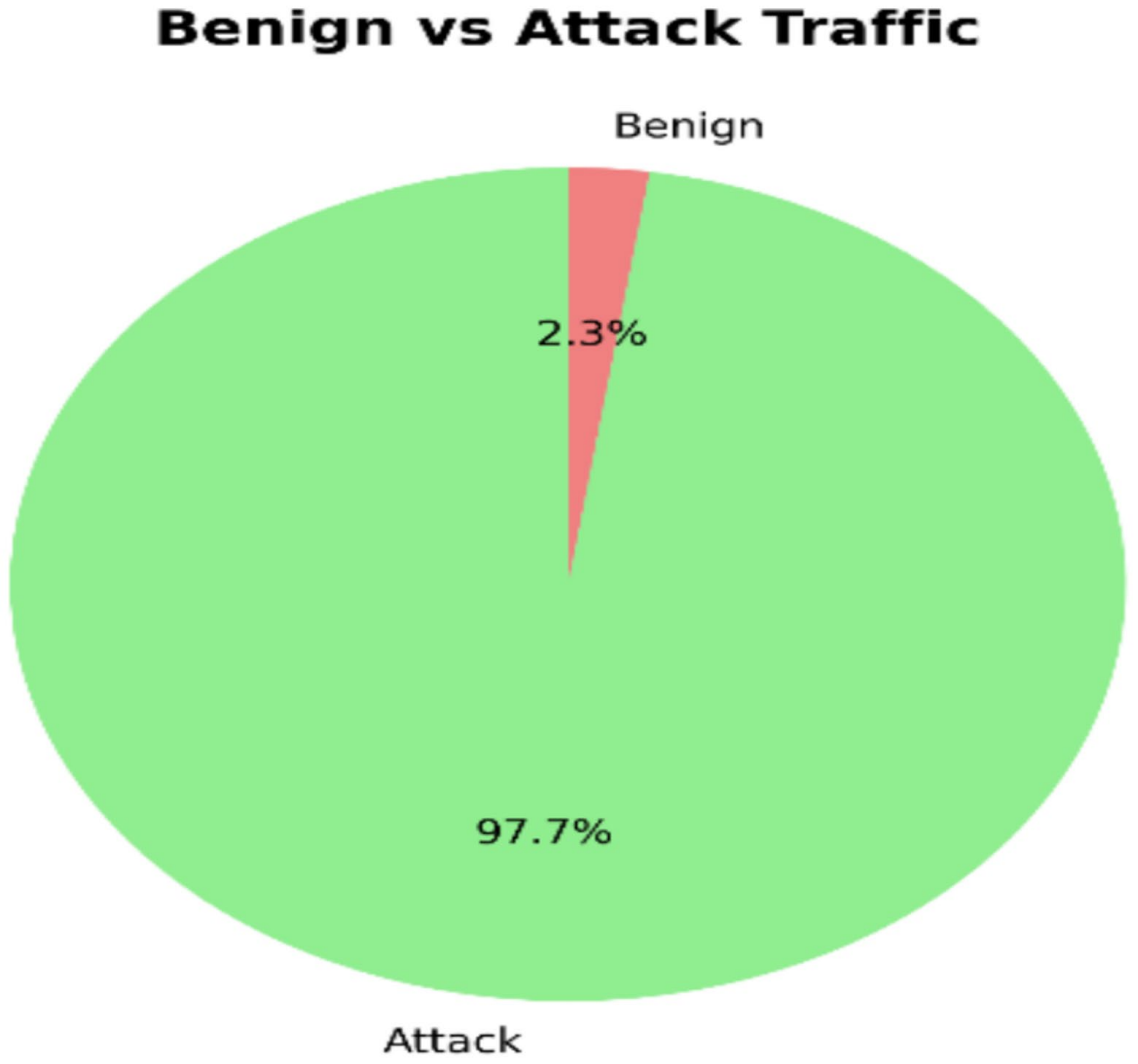
Benign vs. attack traffic.

A pie chart of attack traffic (97.67%) and benign traffic (2.33%) graphically illustrates the dominance of malicious activity in the data, placing emphasis on proper class imbalance treatment in the detection models (Figure 5). The benign attack are attacks traceable to normal, legitimate network activity for instance file downloads, browsing, and application Programming Interface (API) requests from the users while the attack traffic are attacks due to malicious activity designed to harm, exploit, or intrude into a system, for instance, DDoS, Structured Query Language (SQL) injection, brute-force login amongst others. The result presented in Figure 5 indicates the dominance of attack traffic which is an evidence of system’s compromise.

**Figure 6:**
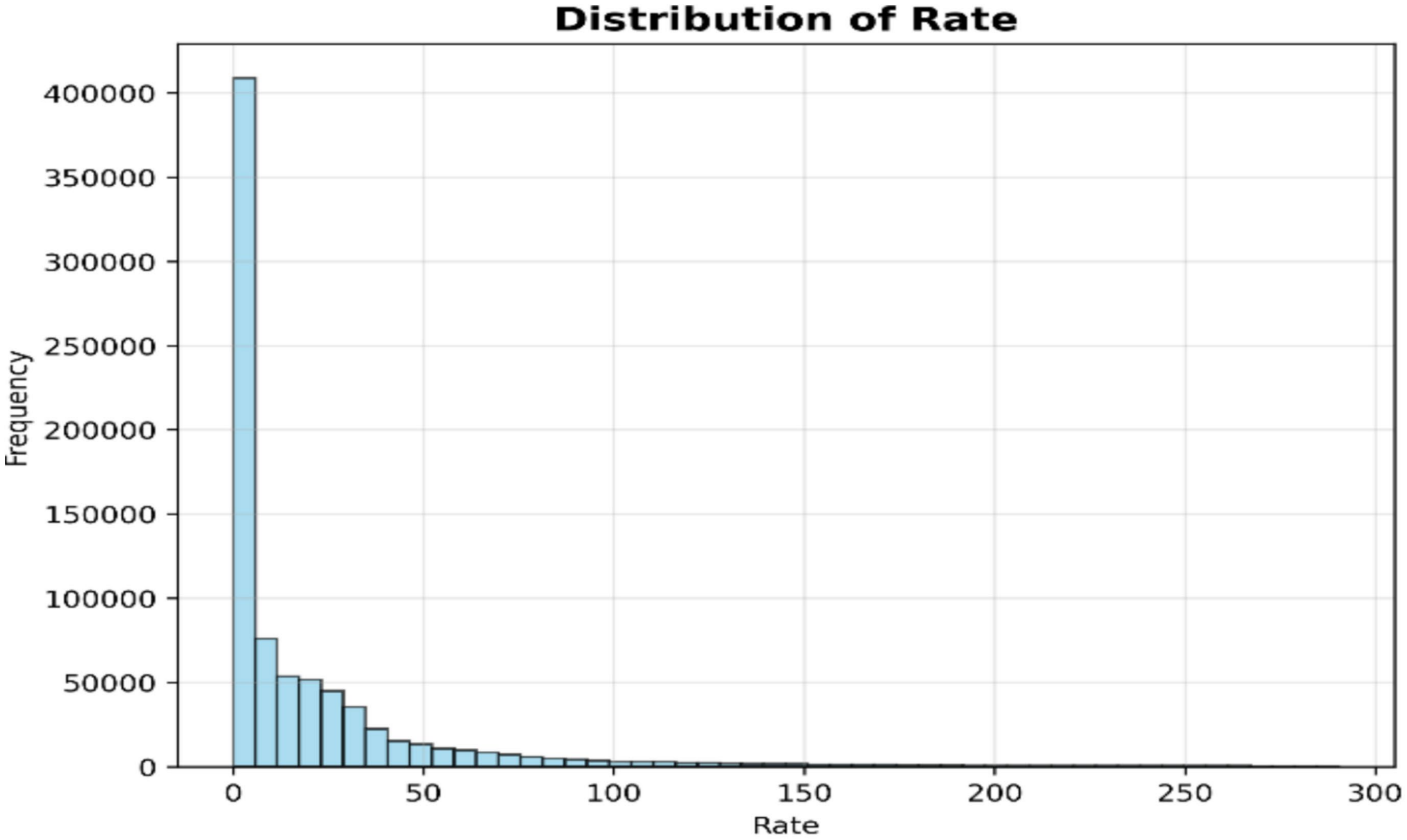
Distribution of rate of attack.

A histogram of the rate attribute, via a histogram, displays the frequency of the different packet transmission rates (Figure 6). This enables the visualization of anomalous patterns, e.g., very high traffic rates that could be indicative of impending attacks.

### 4.3 Model training and performance

Performance metrics on the test set (209,715 samples) for the individual models are summarized in Tables 5–7.

**Table 5:** Performance of Decision Tree model.

Attack class	Precision	Recall	F1-score	Support
Benign-trafic	0.89	0.95	0.92	903
Brute force	0.60	0.10	0.17	10
DDoS	0.98	0.98	0.98	29,134
DoS	0.97	0.97	0.97	6,916
Mirai	0.97	0.97	0.97	2,298
Other	0.75	0.53	0.62	30
Recon	0.79	0.75	0.77	269
Spoofing	0.82	0.80	0.81	428
Web-based	0.85	0.08	0.15	12

**Table 6:** Performance of Random Forest model.

Attack class	Precision	Recall	F1-score	Support
Benign-traffic	0.90	0.96	0.93	903
Brute Force	1.00	0.00	0.00	10
DDoS	1.00	1.00	1.00	29,134
DoS	1.00	1.00	1.00	6,916
Mirai	1.00	1.00	1.00	2,298
Other	0.85	0.57	0.68	30
Recon	0.84	0.77	0.80	269
Spoofing	0.87	0.83	0.85	428
Web-based	1.00	0.00	0.00	12

**Table 7:** Performance of the SVM model.

Attack class	Precision	Recall	F1-score	Support
Benign-traffic	0.91	0.94	0.93	903
Brute Force	0.55	0.05	0.09	10
DDoS	0.99	0.99	0.99	29,134
DoS	0.98	0.98	0.98	6,916
Mirai	0.99	0.99	0.99	2,298
Other	0.80	0.57	0.67	30
Recon	0.85	0.70	0.76	269
Spoofing	0.90	0.78	0.84	428
Web-based	0.95	0.08	0.15	12

The initial dataset employed comprises of 34 distinct attack types. However, the aggregation method was employed to group the attacks into nine categories (listed in Table 5) for effective modelling and evaluation. These include: Benign traffic, Brute Force, DDoS, DoS, Mirai, Other, Recon, Spoofing, and Web-based. The grouping was based on attack taxonomy similarity, putting into consideration the goal of the attack, behavioral patterns of network and features of protocol.

Precisely, attacks with sharing features or mode of operation and detection were grouped together. For instance, multiple flooding-based attacks were grouped under the “DDoS**”** or “DoS” based on their traffic distribution features while attack variants relating to identity theft or credential exploitation were grouped under the **“**Brute Force.” Furthermore, low-frequency and highly specific attack types were grouped under “Other.”

The grouping helps to mitigate severe class imbalance as many attack types had low sample counts that may affect the model’s performance and generalization. It also improves the practical application of the findings in this study especially in the development of intrusion systems that can detect attacks and group them into families rather than detecting individual variants. Additionally, aggregation reduces the noise between highly similar attack subclasses, thus, improving the model’s performance.

#### 4.3.1 Decision tree performance

As shown in Table 5, Decision Tree model achieved the highest accuracy at 99.36% and best performs when it detects dominant attack categories like DDoS-ICMP_Flood (recall: 1.00) and DDoS-UDP_Flood (recall: 1.00). Its capacity to model complex, non-linear patterns and interactions within features well places it appropriately for IoT traffic’s complex patterns. Its performance on minority classes, Backdoor_Malware (recall: 0.53) and SqlInjection (recall: 0.38), indicates struggle with limited training instances.

#### 4.3.2 Random Forest performance

According to Table 6, Random Forest, with a precision of 99.27%, applies ensemble learning to reduce overfitting and performs very well on principal classes but is poor in minority classes like Command Injection (recall: 0.05) and Sql Injection (recall: 0.00), showing The influence of class distribution imbalance on ensemble methods.

#### 4.3.3 SVM performance

According to Table 7, the SVM model, limited to a 50,000-sample training set for computational reasons, achieved a classification accuracy of 80.08%. Its poorer performance on minority classes (e.g., Backdoor_Malware recall: 0.00) suggests that the RBF kernel and reduced dataset fail to capture fully the complexity of the dataset.

Table 8 presents the comparative analysis of the three machine learning models employed for intrusion detection in IoT devices. The results summarized in Table 7 demonstrates the overall performance of the fashions hired on this take a look at for intrusion detection metrics.

**Table 8:** Summary of key findings.

Model	Accuracy (%)	Precision (%)	Recall (%)	F1-Score (%)
Decision Tree	99.36	99.35	99.36	99.35
Random Forest	99.27	99.26	99.27	99.26
SVM	80.08	80.10	80.08	80.09

The Decision Tree achieved the highest accuracy at 99.36%, excelling in detecting dominant attack types such as DDoS-ICMP_Flood (recall: 1.00) but struggling with minority classes like Backdoor_Malware (recall: 0.53). The Random Forest **r**ecorded a slightly lower accuracy of 99.27%, performing well on major attack types but poorly on rare classes, such as Sql Injection (recall: 0.00) while SVM achieved lower predictive performance with an accuracy of 80.08%, primarily due to computational constraints and constrained training configuration, as it was trained on a subset of 50,000 samples. It also struggled with minority classes. Feature importance analysis, derived from the Random Forest model, identified key features such as Inter-Arrival Time (IAT, importance: 0.2576) and Total Size (importance: 0.0776) as critical for distinguishing malicious from benign traffic. The study also highlighted a significant challenge: all models exhibited reduced performance on minority classes because of the dataset’s class imbalance, where dominant attack types like DDoS overshadowed rare attacks like Uploading_Attack.

**Figure 7:**
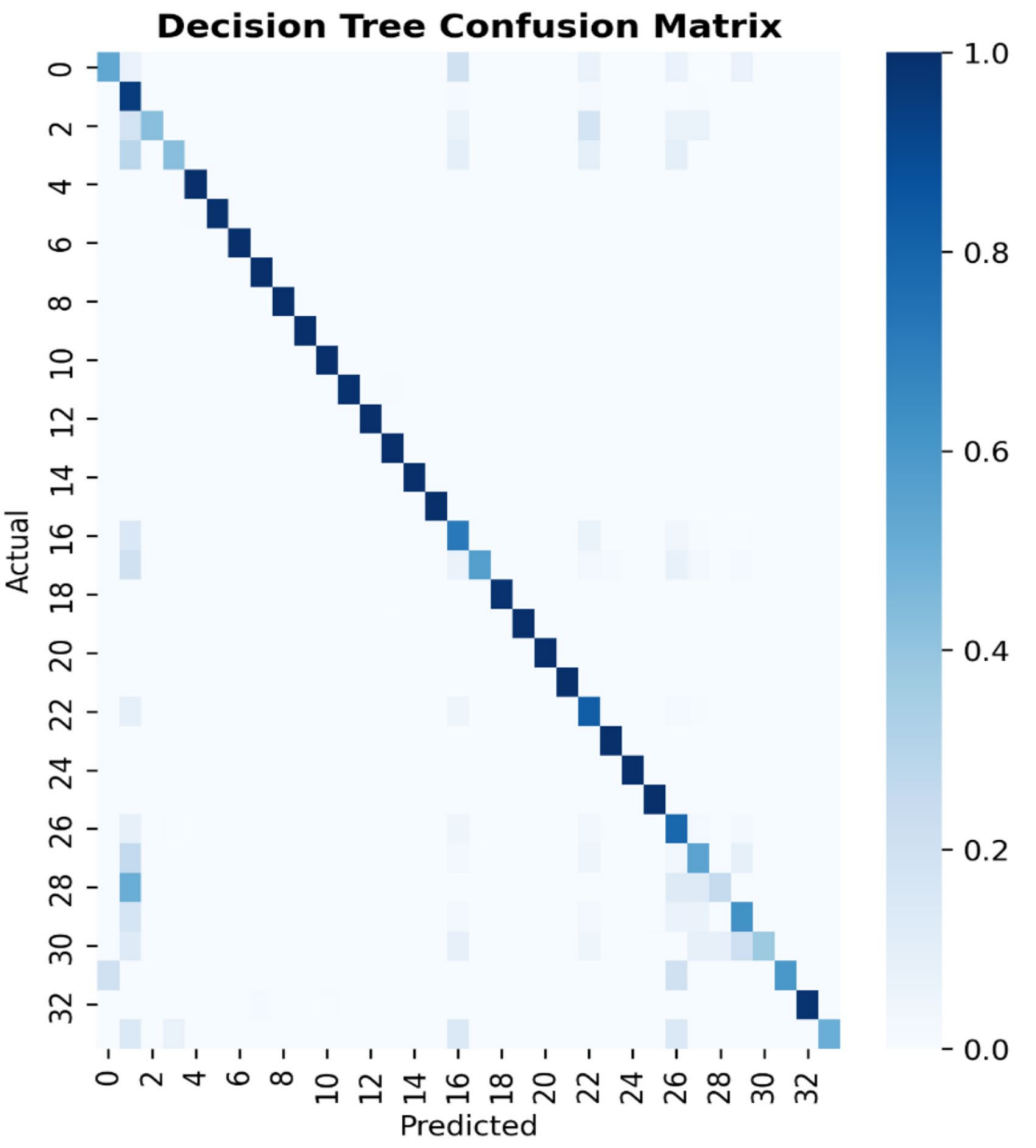
Decision Tree confusion matrix.

**Figure 8:**
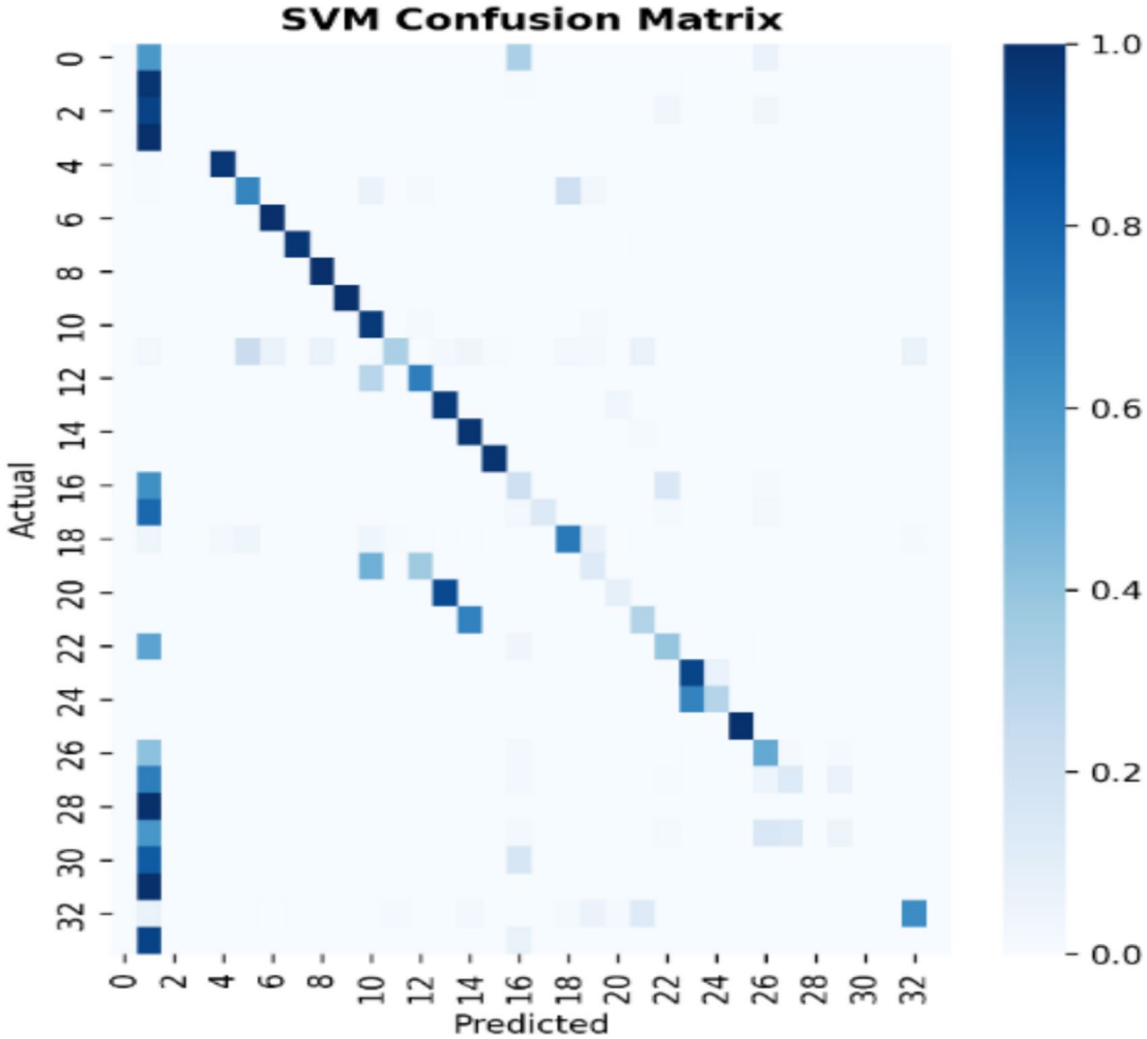
SVM confusion matrix.

**Figure 9:**
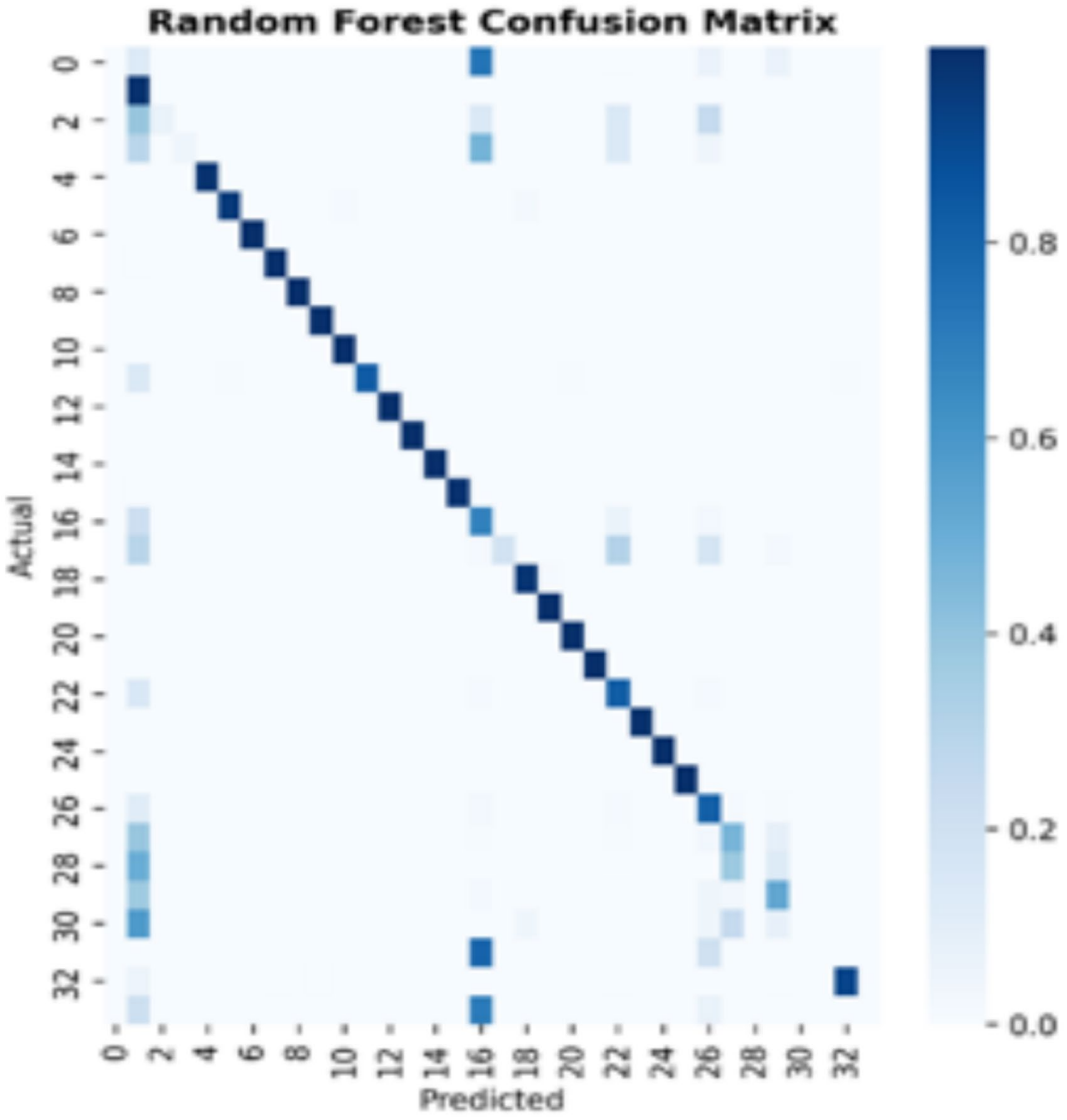
Random forest confusion matrix.

**Figure 10:**
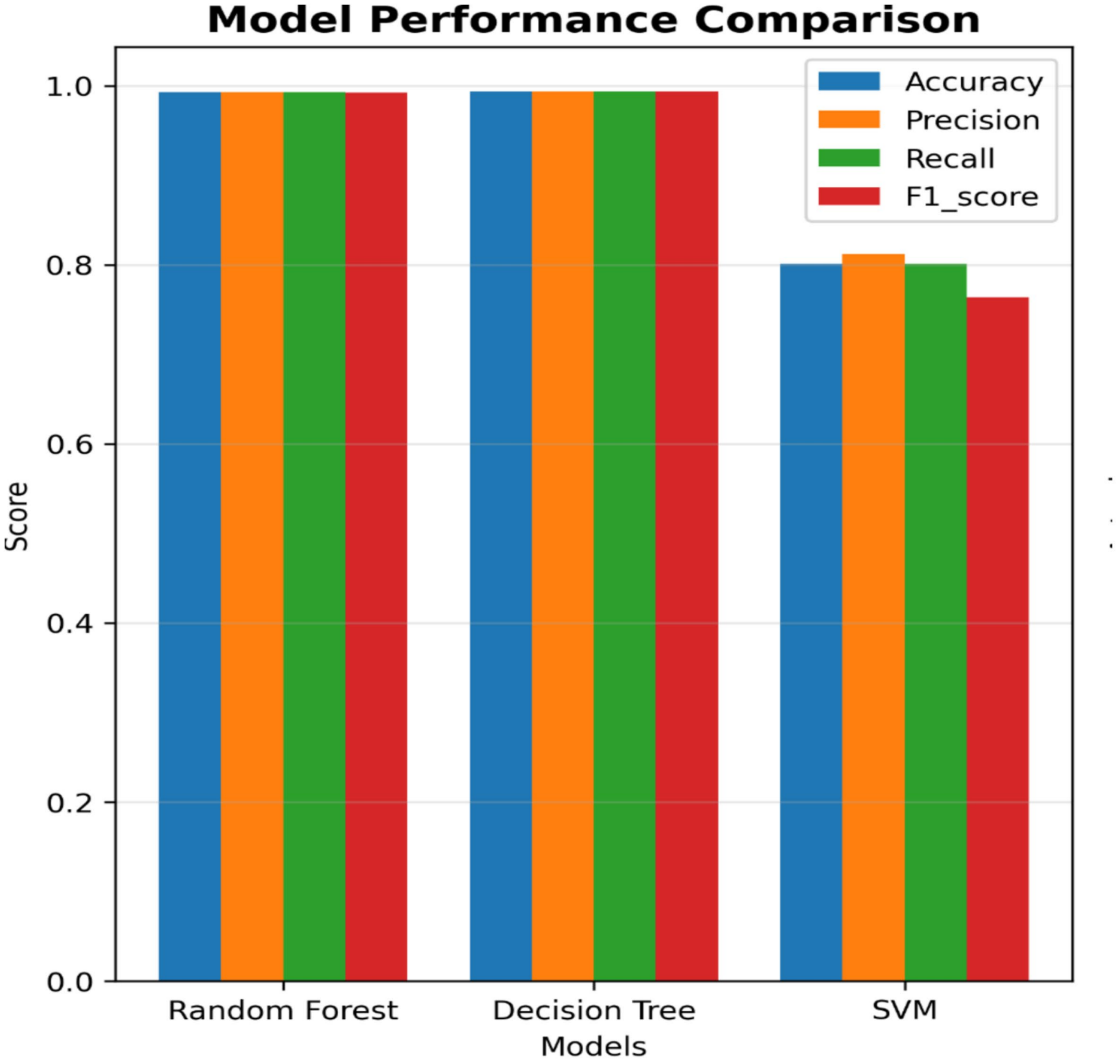
Models’ performance comparison.

Figures 7–9 present the confusion matrices for the DT, SVM and RF ML models employed, respectively. The number of correct classifications are the data points that fall on the diagonal line represents the correct classifications while those that falls off the diagonal line depicts incorrect classifications. It can be seen from the figures that majority of the data points are correctly classified for the DT and RF models with minimal data points falling off the diagonal line compared to the SVM that has some data points outside the diagonal line. This implies that the DT and RF models excel for intrusion classification over SVM in this study. Figure 10 provides a comprehensive visualisation of model performance, including a bar chart comparing accuracy, precision, recall, and F1-score, and confusion matrices for each model, highlighting classification errors. As earlier indicated the Figure further lends credence to the fact that the DT and RF models excel for intrusion classification over SVM in this study.

### 4.4 Feature importance

The Random Forest model’s feature importance analysis identifies key attributes driving intrusion detection, as shown in Table 9.

**Table 9:** Top 10 important features.

Rank	Feature	Importance	Description
1	IAT	0.2576	Inter-arrival time between packets
2	Tot size	0.0776	Total packet size in flow
3	Min	0.0654	Minimum packet size in flow
4	syn_count	0.0541	Number of SYN flags in flow
5	fin_flag_number	0.0488	Presence of FIN flag
6	AVG	0.0429	Average packet size in flow
7	Tot sum	0.0422	Sum of packet sizes in flow
8	fin_count	0.0396	Number of FIN flags in flow
9	Protocol type	0.0385	Network protocol (e.g., TCP, UDP)
10	syn_flag_number	0.0380	Presence of SYN flag

The IAT feature’s high importance reflects its role in detecting rapid packet transmission in attacks like DDoS floods. Tot size and Min help identify anomalous packet sizes, while syn_count and fin_flag_number are critical for TCP-based attacks like SYN floods. These findings align with literature emphasising temporal and size-based features for intrusion detection.

### 4.5 Error analysis

Despite the high overall accuracy of the models, they struggled with minority classes. For instance, the DT model, the Backdoor_Malware has a recall of 0.53, with only 8 of 15 instances correctly classified and the Sql Injection has a recall of 0.38, detecting 9 of 24 instances. Also, the Recon-PingSweep: has a recall of 0.25, identifying only 2 of 8 instances. These errors, evident in the confusion matrix (Figure 7), stem from class imbalance, where minority classes have insufficient samples for robust learning. Potential solutions include (a) Oversampling Using Synthetic Minority Oversampling Technique (SMOTE) to create artificial samples (b) Class Weighting Adjusting model weights to prioritise minority classes and (c) Anomaly Detection Combining classification with unsupervised anomaly detection to identify rare attacks as outliers.

### 4.6 Computational performance values

Table 10 presents the computational performance values after the successful training of the dataset using the selected models. As shown in the Table, the decision tree has the lowest approximate training time, latency and peak RAM due to the fact that it is a fast single model. The Random Forest has the second has approximate training time and latency higher than that of decision tree but lower compared to SVM because it comprises of parallel tree building which is heavy with large dataset employing a quadratic scaling which makes it slow even on smaller subset due to multiple trees voting. The SVM has kernel distance calculations with a heavy kernel matrix memory which contributes too high training time and latency (time delay between the feeding of the into the model and the generation of the output).

**Table 10:** The computational performance values for the selected models.

Model	Approximate training time (mins)	Approximate latency (ms)	Approximate peak RAM (GB)
Decision Tree	4.08	0.30	2.0
Random Forest (100 trees)	31.22	3.00	8.0
SVM (50 k samples, RBF kernel)	66.34	4.01	6.0

## 5 Conclusion and recommendations

The study achieved its goals by developing and comparing three machine learning models for IoT intrusion detection. The Decision Tree model proved most effective with an accuracy of 99.36%, followed by Random Forest with 99.27%. These findings validate the potential of tree-based models in mitigating the multidimensional and complicated nature of IoT network traffic data. Overall assessment, backed by metrics such as recall, precision and F1-score, created a sound comparison of model performance, capable of fulfilling the objective of selecting the most appropriate model for IoT IDS. However, despite the high overall accuracy of these models, they struggled with minority classes. The high accuracy of the DT and RF models indicates their applicability to protect IoT networks from a vast variety of cyber attacks. The interpretability of the DT, whereby decision paths are interpretable by cybersecurity analysts, is what renders it of specific worth for practical use. Its capacity, for example, to detect popular attacks such as DDoS-ICMP_Flood and Mirai botnet attacks with efficacy aligns with increasing demands for security in IoT settings.

These results are particularly pertinent considering the increasing volume of IoT attacks. Yet, the research also demonstrated a critical limitation: the reduced efficiency of the models in identifying minority attack classes caused by class imbalance in the dataset. This is a relevant issue because infrequent attacks, like Backdoor_Malware or Sql Injection, can be a sign of an advanced threat with potentially high impact if not detected. This limitation calls for the development of sophisticated methods for handling class imbalance so that IDS can defend against both frequent and infrequent threats. The study advances IoT security by a comparative analysis of a heavy dataset. The findings provide helpful insights that may be used to protect an IoT network against attacks in various scenarios, such as industrial IoT, smart homes, or critical infrastructure. Furthermore, key features such as IAT and Total Size help focus feature development for Intrusion Detection Systems in the future.

The following are the recommendations to practitioners, researchers, and policymakers in IoT security based on the study findings:

- Deployment of Decision Tree-Based IDS: Decision Tree-based IDS must be prioritized highly for deployment in IoT networks by organizations due to their high accuracy (99.36%) and interpretability. Such models can be integrated into network monitoring systems for the detection and avoidance of common types of attacks effectively.
- Address Class Imbalance: This is to improve detection of infrequent types of attacks, Synthetic Minority Over-sampling Technique (SMOTE), class weights, or anomaly detection must be employed. The effect of these methods is to balance the data set, resulting in better model performance on minority classes like Sql Injection and Backdoor_Malware.
- Optimize for Real-Time Detection: Optimization of Random Forest and Decision Tree models for real-time intrusion detection is required as part of the future work. This includes reducing computational complexity levels to keep pace with high-speed network traffic analysis.
- Continuous Model Updating: As cyber-attacks keep changing, IDS models need provisions for continuous learning. Repeated retraining with new attack patterns and traffic information will guarantee model effectiveness in the long run.

This study is limited to intrusion detection in IoT environment using secondary quantitative dataset, future studies may consider the following:

- Exploration of Ensemble Techniques: Ensemble techniques that fuse multiple models, such as stacking or boosting, may be employed to enhance detection stability and accuracy. For example, the fusion of Random Forest and Decision Tree predictions could provide better performance for minority as well as majority classes.
- Exploration of Deep Learning: While this study focused on traditional machine learning, other deep learning procedures such as convolutional neural networks (CNNs) or recurrent neural networks (RNNs) may be more suited to providing functionality to detect sophisticated attack patterns. Researchers can examine their appropriateness for IoT IDS, particularly zero-day attacks.

## Data Availability

The original contributions presented in the study are included in the article/supplementary material, further inquiries can be directed to the corresponding author/s.
